# Cytidine-phosphate-guanosine oligodeoxynucleotides in combination with CD40 ligand decrease periodontal inflammation and alveolar bone loss in a TLR9-independent manner

**DOI:** 10.1590/1678-7757-2017-0451

**Published:** 2018-05-11

**Authors:** Qian Zhao, Yang Hu, Shu Deng, Pei Yu, Bowen Chen, Zuomin Wang, Xiaozhe Han

**Affiliations:** 1 Capital Medical University, Beijing ChaoYang Hospital, Department of Stomatology, Beijing, China.; 2 The Forsyth Institute, Department of Immunology and Infectious Diseases, Cambridge, MA, USA.; 3 The Secondary Hospital of Tianjin Medical University, Department of Stomatology, Tianjin, China.; 4 Winchester High School, Winchester, MA, USA.; 5 Harvard School of Dental Medicine, Department of Oral Medicine, Infection and Immunity, Boston, MA, USA.

**Keywords:** CpG, Inflammation, Periodontitis, Toll-like receptor 9

## Abstract

Local administration of toll-like receptor 9 (TLR9), agonist cytidine-phosphate-guanosine oligodeoxynucleotide (CpG ODNs), and CD40 ligand (CD40L) can decrease ligature-induced periodontal inflammation and bone loss in wild type (WT) mouse.

**Objective:**

This study aimed to explore whether such effect is dependent on TLR9 signaling.

**Material and Methods:**

Purified spleen B cells isolated from WT C57BL/6J mice and TLR9 knockout (KO) mice were cultured for 48 hours under the following conditions: CD40L, CpG+CD40L, CpG at low, medium and high doses. We determined B cell numbers using a hemocytometer at 24 h and 48 h. Percentages of CD1d^hi^CD5^+^ B cells were detected by flow cytometry. Interleukin-10 (IL-10) mRNA expression and protein secretion were measured by quantitative real-time polymerase chain reaction (qRT-PCR) and by ELISA, respectively. The silk ligature was tied around the maxillary second molars for 14 days, during which the CpG+CD40L mixture or PBS was injected into palatal gingiva on days 3, 6, and 9.

**Results:**

For both WT and TLR9 KO mice, CpG significantly induced B cell proliferation, increased IL-10 mRNA expression and protein secretion of IL-10 but reduced CD1dhiCD5+ B cells population; local injection of CpG+CD40L mixture significantly decreased alveolar bone loss and the number of TRAP-positive cells adjacent to the alveolar bone surface, and significantly increased the gingival mRNA expression of IL-10 and decreased RANKL and IFN-γ mRNA expression.

**Conclusions:**

These results indicated that CpG plus CD40L decreased periodontal inflammation and alveolar bone loss in a TLR9-independent manner in ligature-induced experimental periodontitis.

## Introduction

Periodontitis is characterized by inflammation and alveolar bone loss, which are closely related to bacteria-induced host immune responses.^3^ Conventional therapies for periodontitis focus on clinical methods, such as removal of dental plaque and calculus, reduction of infection and periodontal surgery. Although these treatments are sufficient to treat periodontal diseases in many cases, the host immune response has been sustained in an imbalanced state. Recently, researchers have explored immune mechanisms and treatment strategies associated with periodontitis by looking for novel inflammatory pathways,^8^ regulating host innate and adaptive immune responses,^9^ and exploring immune mechanisms that inhibit alveolar bone loss.^29^

Toll-like receptors (TLRs), a class of immune molecules closely related to periodontitis, consist of at least 13 proteins working as key pathogen recognition receptors (PRRs) in innate immunity system and respond to many kinds of microbial products and injury-induced endogenous products.^13^ Of these receptors, TLR9 is unique for its engagement in both microbial nucleic acids and danger-associated molecular patterns (DAMPs), and communication with other TLRs.^5^ TLR9 can activate pro- and/or anti-inflammatory signaling pathways and mediate inflammatory responses in periodontitis pathogenesis.^8^ In another study using oral gavage model of periodontitis, Kim, et al.^11^(2005) found that TLR9 Knockout (KO) mice show better resistance to periodontal inflammation and TLR9 can regulate TLR2- and TLR4-triggered inflammation by downstream signaling pathways.

TLR9 is not only activated by microbial nucleic acids, but also by synthetic cytidine-phosphate-guanosine (CpG) DNA motifs.^11^ At the molecular level, internalization of CpG activates TLR9 mediated sequence recruitment signals of MyD88, interleukin receptor associated kinase (IRAK) and TNF-receptor associated factor 6 (TRAF6), which activate downstream transcription factors (NF-κB and AP-1) causing the induction of proinflammatory cytokines.^9^ Some studies have suggested that preventive therapy in mice using synthetic CpG can protect them against diverse infections.^21^ ^,^ ^22^ Mouse B cell was one of the first definite cell types which has been proved to directly respond to immunostimulatory CpG-containing DNA sequences *in vitro* and *in vivo.* ^12^ ^,^ ^17^ Our recent study has also suggested that local administration of CpG plus CD40L can induce B cell IL-10 competency thus decreasing inflammation and bone loss in ligature-induced experimental periodontitis in WT mice.^29^

CD40 ligand (CD40L), expressed by activated T cells with its receptor CD40 on B cells, is another important costimulatory activation pathway in B cells. The activation of CD40 on B cells also activates NF-κB and the signal transduction modules overlap with those in TLR9 signaling. CD40/CD40L interaction is necessary for a variety of reactions in a cell-dependent humoral immune response, including B cell survival and proliferation, germinal center and memory B cell formation, Ab isoform conversion and maturation, and upregulation of molecules such as CD23, ICAM-1 and CD80.^28^

Although CpG is presumed to act through TLR9, some evidence suggested that CpG may play an immunomodulatory role in a TLR9-independent manner.^2^ ^,^ ^23^ ^,^ ^27^ Whether CpG plus CD40L reduce bone loss through TLR9 signaling in experimental periodontitis was not addressed yet. This study aimed to explore the effects of CpG in combination with CD40L on B cell expansion and function, and whether CpG plus CD40L reduce periodontitis inflammation and alveolar bone loss in a TLR9-independent manner.

## Material and methods

### Animals

Wild type C57BL/6J mice and TLR9 KO (Tlr9^M7Btlr^/Mmjax) mice, which were from 8 to 10 weeks old and were maintained under pathogen-free conditions, were from The Jackson Laboratory (Bar Harbor, ME). For experimental ethical approval, the Institutional Animal Care and Use Committee of the Forsyth Institute approved the protocol.

### B cell isolation and culture

WT mice and TLR9 KO mice were sacrificed in CO_2_ chamber and spleens were harvested. All cell culture supplies (USA Scientific) were RNase, DNase, and pyrogen free. Culture experiments were carried out in designated biological safety cabinet and work bench, which was stringently maintained clean and disinfected all time. Spleens were softly processed through metal mesh in Iscove's modified Dulbecco's medium (IMDM; Gibco). Red blood cells were removed with ACK lysis buffer (Life Technologies) and single cell suspension was obtained by filtering the spleens through a 40-μm-mesh-size cell strainer screen. B cells were isolated via magnetic columns (Miltenyi Biotec) by a pan-B cell isolation kit (Miltenyi Biotec). Purified B cells (>95%, 1×10^6^ cells/well) were cultured in a 96-well culture plate with 200 μl of complete medium [IMDM containing 10% fetal bovine serum, 2.5 μg/ml amphotericin B (Fungizone), 0.1% 2-mercaptoethanol, 2 mM L-glutamine, 100 U/ml penicillin, 100 U/ml streptomycin] for 24 h and 48 h with or without CD40L (eBioscience) or CpG (ODN 2006 oligodeoxynucleotides; 5'-TCCATGACGTTCCTGATGCT-3'; Hycult Biotech) stimulation. Purified B cells were divided into six groups (n=4 WT mice, or TLR9 KO mice *per* group): control group (none stimulation), CD40L group (0.1 μg/ml CD40L), CD40L (0.1 μg/ml) + CpG (1 μM CpG) group, CpG-Low group (0.1 μM CpG), CpG-Med group (1 μM CpG) and CpG-High group (10 μM CpG).

### ELISA assay

We used Mouse IL-10 enzyme-linked immunosorbent assay (ELISA) Max Standard kit (BioLegend) to measure the IL-10 secretion levels in the supernatants of cultured B cells. The assay was performed in duplicate and a standard curve was generated. The absorbance (450 nm) was detected in a microplate reader (BioTek), and the IL-10 concentration (pg/ml) was analyzed according to the standard curve.

### Flow cytometry analysis

B cells were rinsed with cell staining buffer and incubated with blocking buffer including anti-mouse CD16/32 antibody (Ab) after they were cultured for 48 hours. Then B cells were stained by allophycocyanin-labeled anti-mouse CD5 Ab (BioLegend) and phycoerythrin-labeled anti-mouse CD1d (BioLegend). Data were collected on a FACSAria flow cytometer (BD Biosciences) and analyzed by FlowJo software (TreeStar, Inc.).

### Experimental periodontitis animal model

A modified mouse model of ligature-induced experimental periodontitis was generated based on method previously described.^1^ Six WT mice and 8 TLR9 KO mice were randomly selected for each group. On day 0, the silk thread of size 7-0 (Fisher Scientific) were ligated around both maxillary second molars in each mouse and remained for 2 weeks. The palatal gingiva on the left side was injected with a CpG+CD40L mixture (0.1 μg/ml of CD40L + 40 μM CpG) and that on the right side was injected with vehicle control (PBS). Insulin syringes (Gauge 31, 3/10cc, BD Biosciences) were used for the injection. To perform the injection accurately, the tip of each needle was blunted to ensure that its tip was embedded in the gingiva during the procedure. On days 3, 6, and 9, CD40L+CpG or PBS was injected into the palatal gingiva of maxillary second molars of each mouse. We performed the whole procedures of ligature and injection using an optical microscope (S6D Stereozoom, Leica).

### Sample preparation

On day 14, all the mice were sacrificed by CO_2_ inhalation. Four WT mice and 4 TLR9 KO mice from each group were randomly selected for bone morphometric analysis. After the skin and muscle were removed from harvested maxillae, palatal gingival tissues around the left and right second molars were collected under a surgical microscope. The gingival tissues were stored in −80°C for detecting mRNA expression of inflammatory cytokines. Then the maxillae were defleshed by a dermestid beetles colony. The maxillae harvested from the remaining mice were processed and fixed with 10% paraformaldehyde for 12 hours. Then the maxillae were decalcified in 10% EDTA for 3 weeks at 4°C with agitation. After demineralization, all tissue samples were immersed in 10% and 30% sucrose solution and then embedded in OCT solution (Tissue-Tek). We cut the frozen samples in 8 μm along the tooth crown-root plane using Cryostat and then we gathered them on Superfrost-plus slides (Fisher Scientific) for histological analysis.

### Real time quantitative PCR

**Figure 1:**
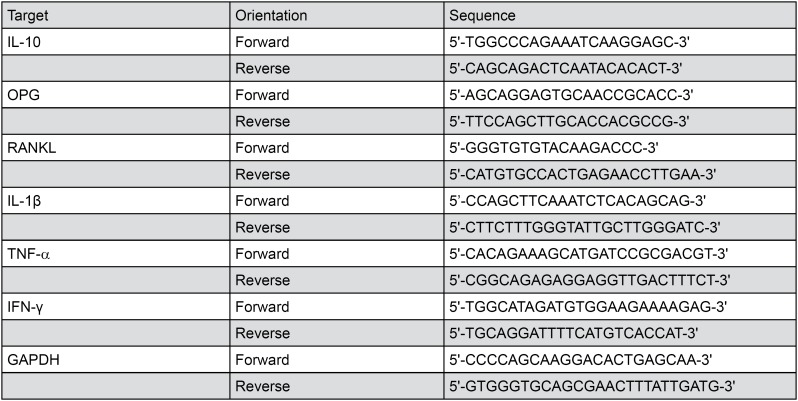
Primers and sequences used for qRT-PCR. Mouse IL-10, OPG, RANKL, IL-1β, TNF-α, IFN-α and GAPDH primers for qRT-PCR are listed

For *in vitro* experiment (*n=*4 for WT mice or TLR9 KO mice), B cells cultured for 48 h were collected and lysed with lysis buffer for RNA extraction. For *in vivo* experiment (*n=* 4 for WT mice or TLR9 KO mice), gingival tissues stored in −80°C were defrosted and homogenized by a tissue homogenizer (Omni). We extracted total RNA of cultured B cells or each homogenized gingiva sample using a PureLink RNA mini kit (Life Technology). We obtained cDNA using a SuperScript II reversed transcriptase kit (Life Technology). The IL-10 mRNA expression from cultured B cells, and mRNA expression of gingival IL-10, osteoprotegerin (OPG), receptor activator of nuclear factor-κB ligand (RANKL), IL-1β, TNF-α and IFN-α were detected by qRT-PCR via LightCycler SYBR green I master mix and a LightCycler 480 instrument system (Roche). All the sequences of primers are listed in Figure 1. The glyceraldehyde-3-phosphate dehydrogenase (GAPDH) gene was used as internal control.

### Bone morphometric analysis

The defleshed maxillae were bleached with 3% hydrogen peroxide for 8 hours and stained with 1% toluidine blue. The degree of alveolar bone loss was measured by Nikon microscope (SMZ745T, Nikon Instruments Inc). Images of the palatal alveolar bone of each maxilla were acquired, and the specific area presenting alveolar bone loss was measured by Image J software (NIH). The defined method of the area has been previously described.^16^ It was formed longitudinally from the cementoenamel junction to the alveolar bone crest and transversely from the distal area of the first maxillary molar to the mesial area of the third maxillary molar. The results of bone loss area were expressed in square millimeters (*n=*3 for WT mice, *n* =4 for TLR9 KO mice).

### Tissue histological analysis

A tartrate-resistant acid phosphatase (TRAP) staining method was used for tissue histological analysis. 8 μm-thick sample sections were stained with an acid phosphatase kit (378A, Sigma) and counterstained with hematoxylin. Histological images were captured by a light microscope with a magnification of 20× (DMLS, Leica) and TRAP positive cells with three or more nuclei were regarded as osteoclasts. An area from gingival papilla to root apical between the second molar and adjacent molars was applied to each sample. Osteoclast numbers at the infiltrated connective tissue within the area abovementioned were counted and the values were averaged for statistical analysis (*n=*4 for WT mice or TLR9 KO mice).

### Statistical analysis

Results of cultured B cell numbers, CD1d^hi^ CD5^+^ B cell percentages, levels of IL-10 gene expression and protein secretion, bone loss square, TRAP positive cell numbers and levels of inflammatory cytokines mRNA expression were represented by mean±SD. Unpaired Student's t-test was used to analyze differences on B cell numbers, CD1d^hi^ CD5^+^ B cell percentages, levels of IL-10 gene expression and protein secretion between control group and different treatment groups in WT mice and TLR9 KO mice *in vitro.* Paired Student's t-test was used to analyze the variances on the alveolar bone loss area, levels of inflammatory cytokine gene expression and the trap positive cell numbers of tissues between control side and treatment side in WT mice and TLR9 KO mice *in vivo*. A p value of <0.05 was considered statistically significant.

## Results

### CpG induced B cell proliferation on WT mice and TLR9 KO mice *in vitro*

**Figure 2:**
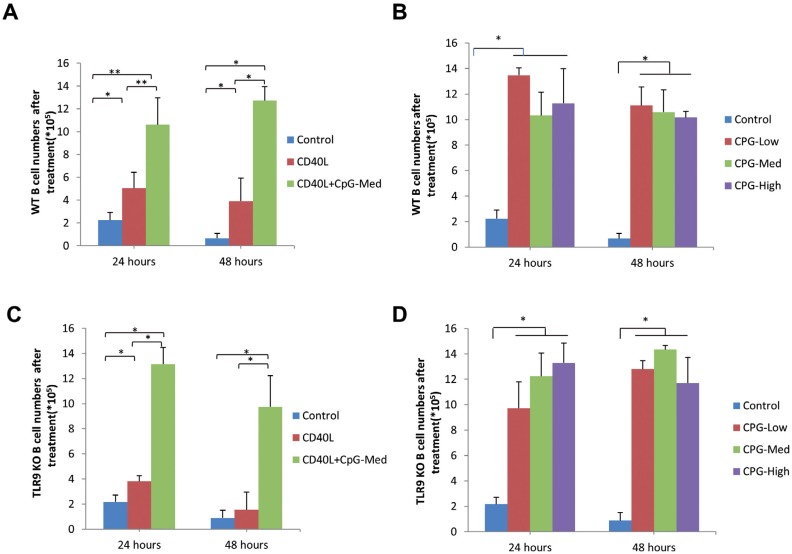
CpG induced proliferation of spleen B cells from WT and TLR9 KO mice. Purified spleen B cells separated from C57/BL6J (WT) mice were cultured 24 h and 48 h with (A) CD40L (0.1 μg/ml), CpG (1 μM)+CD40L (0.1 μg/ml), (B) CpG-Low (0.1 μM), CpG-Med (1 μM) and CpG-High (10 μM) (mean±SD, n=4 WT mice per group, *p<0.05, **p<0.01). Purified spleen B cells separated from TLR9 KO mice were cultured 24h and 48h with same treatment group including CD40L, CpG+CD40L(C) and different doses of CpG (D) (mean±SD, n=4 TLR9 KO mice *per* group, *p<0.05, **p<0.01)

We determined B cell numbers using a hemocytometer after stimulation with CD40L and CpG for 24 h and 48 h in WT mice (Figures 2A, 2B) and TLR9 KO mice (Figures 2C, 2D). Regarding either type of mice, B cell numbers had a significant increase at two time points after stimulated by CD40L (0.1 μg/ml) + CpG (1 μM) (*p*<0.05). CD40L alone significantly increased B cell proliferation compared to control group (*p*<0.05) in WT mice 24 h and 48 h, and in TLR9 KO mice at 24 h. Also in each type of mice, B cell numbers were significantly increased at two time points compared to control group after stimulation with CpG-Low (0.1 μM), CpG-Med (1 μM) and CpG-High (10 μM) (*p*<0.05), but such increase in B cell numbers had no significant difference among different CpG-treated groups (*p*>0.05). Moreover, B cell numbers had no significant difference at two time points between WT mice and TLR9 KO mice (*p*>0.05).

### CD1d^hi^CD5^+^ B cell expansion was induced by CD40L and inhibited by CpG *in vitro*

**Figure 3:**
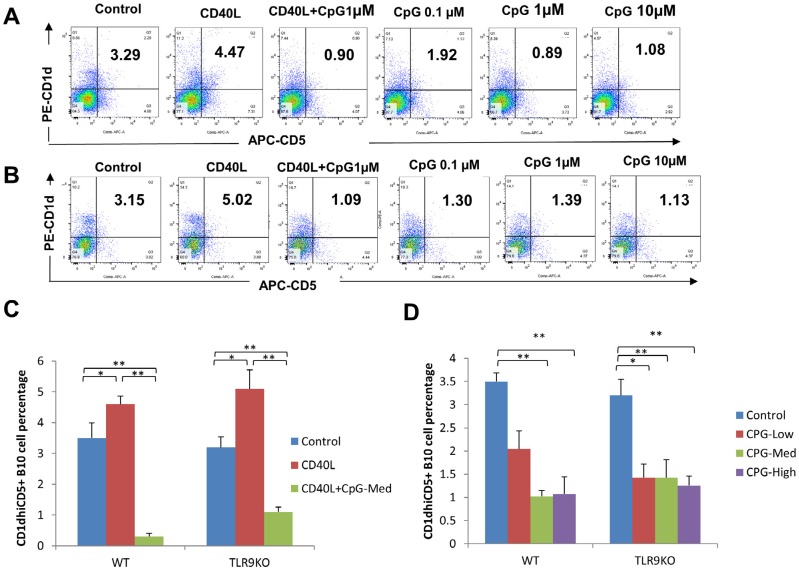
CpG reduced CD1d^hi^CD5^+^ B cell percentages in spleen B cells from WT and TLR9 KO mice. The percentages of CD1d^hi^CD5^+^ B cells were detected by flow cytometry from purified spleen B cells separated from WT mice (A) and TLR9 KO mice (B) cultured with CD40L (0.1 μg/ml), CpG (1 μM)+CD40L (0.1 μg/ml), CpG-Low (0.1 μM), CpG-Med (1 μM) and CpG-High (10 μM) for 48 h. The percentages of CD1d^hi^CD5^+^ B cells were analyzed and compared between groups including control, CD40L and CpG+CD40L (C) and groups including CpG-Low, CpG-Med and CpG-High (D) (mean±SD, n=4 WT mice or TLR9 KO mice *per* group, *p<0.05, **p<0.01)

Figure 3 showed flow cytometry results of CD1d^hi^CD5^+^ B cell percentages in WT mice (Figure 3A) and TLR9 KO mice (Figure 3B) in the different groups abovementioned. In both types of mice, CD1d^hi^CD5^+^ B cell percentages were significantly lower in CpG+CD40L treatment group compared to control group or CD40L treatment group (*p*<0.01). In CD40L treatment group, CD1d^hi^CD5^+^ B cell percentage was higher than control group in WT and TLR9 KO mice (*p*<0.05) (Figure 3C). Moreover, CpG-Med and CpG-High significantly inhibited CD1d^hi^CD5^+^ B cell expansion compared to control group (*p*<0.01) in both WT and TLR9 KO mice. Similar inhibitory effect was observed in CpG-Low group in TLR9 KO mice (*p*<0.05) but not in WT mice (Figure 3D).

### IL-10 mRNA expression and protein expression were increased by CpG stimulation in B cells from both WT and TLR9 KO mice

**Figure 4:**
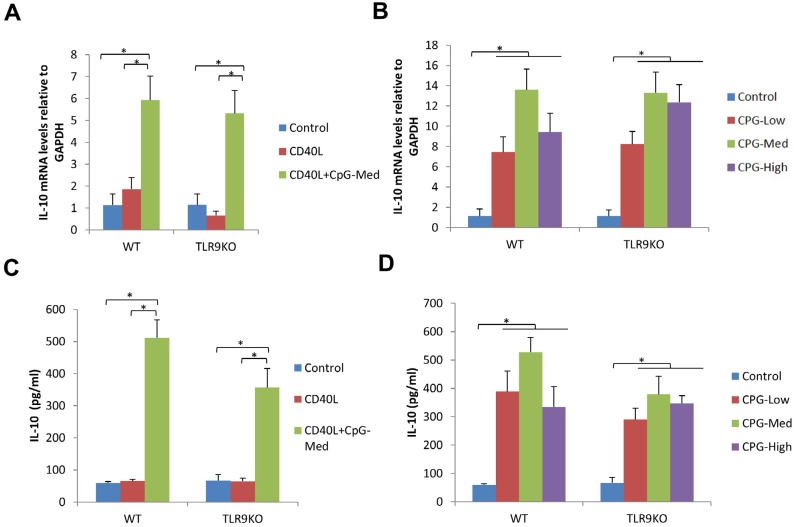
CpG increased IL-10 mRNA expression and protein secretion in spleen B cells from WT and TLR9 KO mice. Purified spleen B cells separated from WT mice and TLR9 KO mice were cultured with CD40L (0.1 μg/ml), CpG (1 μM) +CD40L (0.1 μg/ml) for 48 h. The levels of IL-10 mRNA expression (A) and protein secretion (C) were detected by qRT-PCR and ELISA, respectively (mean±SD, n=4 WT mice or TLR9 KO mice per group, *p<0.05, **p<0.01). Purified spleen B cells separated from WT mice and TLR9 KO mice were cultured with CpG-Low (0.1 μM), CpG-Med (1 μM) and CpG-High (10 μM) for 48 hours. The levels of IL-10 mRNA expression (B) and protein secretion (D) from cultured B cells were detected by qRT-PCR and ELISA, respectively (mean±SD, n=4 WT mice or TLR9 KO mice per group, *p<0.05, **p<0.01)

The levels of IL-10 mRNA and protein expression were measured from the groups abovementioned. In B cells from both WT and TLR9 KO mice, the levels of IL-10 mRNA expression and IL-10 secretion were significantly increased in CpG+CD40L treatment group compared to control group and CD40L treatment group (*p*<0.05). However, CD40L alone had no significant effect on IL-10 mRNA expression and IL-10 secretion (Figures 4A, 4C). All doses of CpG treatment significantly increased IL-10 mRNA expression and IL-10 secretion compared to control group (*p*<0.05) (Figures 4B, 4D). No significant difference was observed among CpG treatment groups in both WT mice and TLR9 KO mice (*p*>0.05) (Figures 4B, 4D).

### Periodontal alveolar bone loss was inhibited by local administration of CpG+CD40L in both WT and TLR9 KO mice

**Figure 5:**
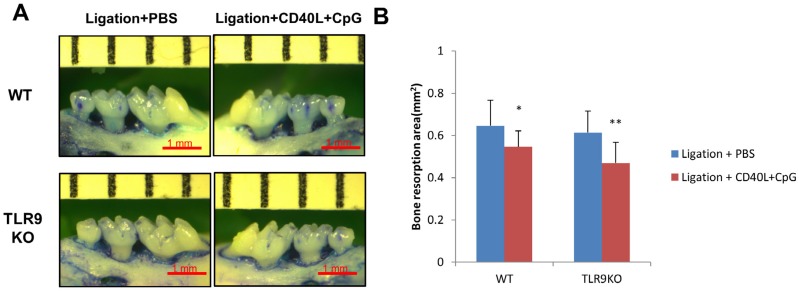
CpG+CD40L reduced bone loss in ligature-induced experimental periodontitis of WT and TLR9 KO mice. Silk ligatures were tied around the maxillary secondary molars on day 0 and injection of CpG (1 μM)+CD40L (0.1 μg/ml) or PBS was performed in palatal gingiva on days 3, 6 and 9 in WT mice and TLR9 KO mice. Maxillae were collected on day 14, and the area of palatal alveolar bone loss around the maxillary secondary molars was pictured (A) and analyzed (B). Data were presented as the bone loss area (square millimeter) determined at a magnification of 30X (means±SD, n=4 WT or TLR9 KO mice *per* group, *p<0.05, **p<0.01, compared with PBS injection group)

Alveolar bone loss at left (CpG+CD40L injection) and right (PBS injection) palatal gingiva were measured in WT mice and TLR9 KO mice of ligature-induced experimental periodontitis model (Figure 5A). Based on the analysis of alveolar bone loss around maxillary second molars, the periodontal bone loss was significantly decreased after treatment with CpG+CD40L (*p*<0.05) compared with control group (PBS treatment side) in both WT mice and TLR9 KO mice (Figure 5B). Moreover, there was no significant differences between WT and TLR9 KO mice on bone loss of CpG+CD40L treated side (*p>0.05*) (Figure 5B).

### The numbers of multinucleated TRAP-positive cells were reduced by local administration of CpG+CD40L in both WT and TLR9 KO mice.

**Figure 6:**
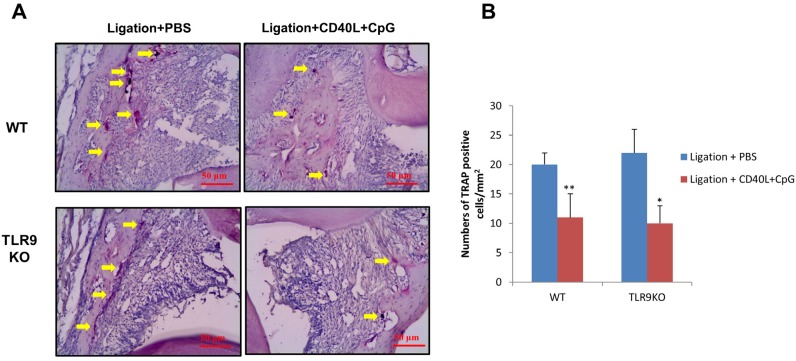
CpG+CD40L reduced TRAP positive cells loss in ligature-induced experimental periodontitis of WT and TLR9 KO mice. Silk ligatures were tied around the maxillary secondary molars on day 0 and injection of CpG (1 μM)+CD40L (0.1 μg/ml) or PBS was performed in palatal gingiva on days 3, 6 and 9 in WT mice and TLR9 KO mice. TRAP staining was performed on periodontal tissues and images were analyzed at a magnification of 200X (A). Bar charts showed the mean numbers of multinucleated TRAP-positive cells along the alveolar bone surface (means±SD, n=4 WT or TLR9 KO mice *per* group, *p<0.05, **p<0.01, compared with PBS injection group)

The osteoclastogenic activities in periodontal gingival tissues were evaluated by tartrate-resistant acid phosphatase (TRAP) staining (Figure 6A). In both WT mice and TLR9 KO mice, the number of multinucleated TRAP-positive cells adjacent to the alveolar bone surface was significantly decreased in CpG+CD40L treatment group compared with control group (*p*<0.05) (Figure 6B).

### Gingival IL-10 mRNA expression was increased and RANKL and IFN-γ mRNA expression was decreased after local administration of CpG+CD40L in WT mice and TLR9 KO mice.

**Figure 7:**
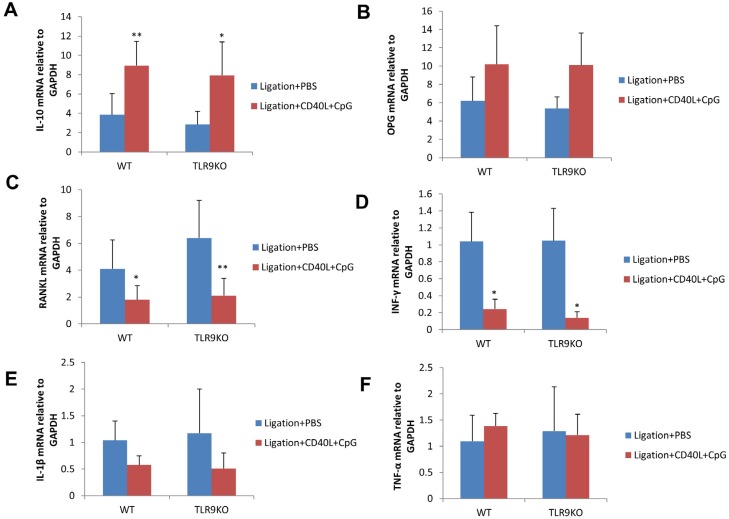
CpG+CD40L increased gingival mRNA levels of IL-10 and decreased gingival mRNA levels of RANKL and IFN-γ in ligature-induced experimental periodontitis of WT and TLR9 KO mice. Silk ligatures were tied around the maxillary secondary molars on day 0 and injection of CpG (1 μM)+CD40L (0.1 μg/ml) or PBS was performed in palatal gingiva on days 3, 6 and 9 in WT mice and TLR9 KO mice. The levels of mRNA expression for IL-10 (A), OPG (B), RANKL (C), IL-1β (D), TNF-α (E), IFN-γ (F) in gingival tissues were detected by qRT-PCR and their relative levels of expression were normalized to the level of GAPDH expression (means±SD, n=4 WT or TLR9 KO mice *per* group, *p<0.05, **p<0.01, compared with PBS injection group)

The levels of gingival mRNA expression of IL-10, OPG, RANKL, IFN-γ, IL-1β and TNF-α were measured by qRT-PCR. In both WT and TLR9 KO mice, the mRNA level of gingival IL-10 expression was significantly increased and the mRNA levels of gingival RANKL and IFN-γ was significantly decreased in CpG+CD40L treatment group compared with control group (*p*<0.05) (Figures 7A, 7C, 7D). No significant difference in the mRNA levels of gingival IL-1β, TNF-α, OPG was observed between the CpG+CD40L treatment group and control group (*p*>0.05) (Figures 7B, 7E, 7F).

## Discussion

CpG demonstrated to be effective as vaccine adjuvant, and thus is indicated for the treatment of cancer, infectious and allergic diseases.^14^ ^,^ ^15^ ^,^ ^26^ It can directly induce the activation and maturation of plasmacytoid dendritic cells (pDCs) and promote differentiation of B cells into antibody-secreting plasma cells. The common recognition is that CpG plays a role in immune regulation, which is dependent on TLR9. Our study consisted in CPG plus CD40L local administration in WT mice and TLR9 KO mice with ligature-induced periodontitis model. We discovered that after a two-week treatment, the effects of CpG+CD40L on decreasing periodontal inflammation and alveolar bone loss had no significant difference between WT mice and TLR9 KO mice. Thus, for the first time, our study suggested that local application of CpG plus CD40L inhibit ligature-induced periodontitis in a TLR9-independent manner.

Some studies have shown that CpG not only can directly co-stimulate mouse and human CD4^+^T cells, but it can also stimulate B lymphocytes via a TLR9-independent mechanism.^4^ ^,^ ^6^ ^,^ ^19^ ^,^ ^23^ These observations were consistent with our *in vitro* results, which showed that CpG alone had similar effects on inducing B cell population and increasing B10 cells activation from both WT and TLR9 KO mice. Moreover, many studies mentioned TLR9-independent CpG signaling in neutrophils, DCs, mouse embryonic stem cells, macrophages, and THP-1 cells.^7^ ^,^ ^10^ ^,^ ^20^ ^,^ ^23^ ^,^ ^27^ Thus, we speculate that CpG modulates B cell expansion and B10 cell activation via a TLR9-independent pathway, which needs further studies as indicated in other types of cells abovementioned.

Furthermore, there are many possible pathways through which CpG plays an immunomodulatory role in periodontitis. Zhu, et al.^30^ (2009) found a nucleic acid-induced signal transduction pathway, named scavenger B1 receptor (SR-B1), which can provide feedback to suppress specific TLR9-independent responses of B cells. They suggested that CpG/SR-B1 triggers calcium entering into primary B lymphocytes through phospholipase Cγ-1-mediated activation of TRPC3 channels and B cells adhering to vascular cell adhesionmolecule-1 (VCAM-1). This kind of CpG-induced, SR-B1 exclusively mediated calcium signals and VCAM-1 adhesion are TLR9-independent, providing a unique perspective on the complex effects of CpG signaling pathway within B cells. Another study suggested that CpG can induce CCN1 secretion and IL-10 release from lung epithelial cells by the BiP/GRP78-Src(Y527)-JNK-Cav-1(Y14) pathway, which can suppress TNF-α, macrophage inflammatory protein 2 (MIP-2) secretion and neutrophil infiltration in the lungs.^18^ In addition, CpG can protect human monocytic cells against HIV-viral protein R (Vpr) induced apoptosis by the calcium-activated JNK pathway in a TLR9-independent manner.^24^ Although pathogenesis and pathological mechanism of periodontitis are different from those diseases, these studies provide us reasons for further investigating the underlying TLR9-independent mechanism of CpG in periodontitis.

CD40L can activate membrane-associated protein CD40 on B cells, inducing maturation of B cells into antibody-producing B cells. It is also crucial for activating regulatory B cells.^25^ Our results showed that CpG alone induced activation of B10 function (IL-10 secretion), however, it also significantly reduced B10 frequency in cultured splenic B cells. In addition, CD40L alone induces B10 expansion but not IL-10 secretion. Thus, the combination of CD40L with CpG will both induce B10 activation and maintain B10 population. The synergy effects were shown in B cells from WT mice *in vitro* and ligation-induced periodontitis model in WT mice in *vivo* in our previous study.^29^ Moreover, the effects of different concentration of CpG+CD40L on decreasing ligature induced periodontitis have been previously studied.^29^ The previous experimental results showed that low concentration of CpG+CD40L (1 uM of CpG+0.1 ug/ml of CD40L) had a higher inhibitory effect on ligature induced periodontitis and alveolar bone loss than that of high concentration of CpG+CD40L (10 uM of CpG+1 ug/ml of CD40L). Thus, low concentration of CpG+CD40L (1 uM of CpG+0.1 ug/ml of CD40L) was selected for our experiments.

In summary, our study investigated the effects of CpG+CD40L on B10 cell population and activation from both WT and TLR9 KO mice, indicating a possible TLR9 independent mechanism of this synergy effect.

## Conclusions

In conclusion, our study demonstrated that local administration of CpG+CD40L can decrease periodontal inflammation and alveolar bone loss in a TLR9-independent mechanism. The unknown mechanism remains to be investigated in the future, which is important to develop immunological interventions for treatments of periodontitis.
